# Food as a tool for learning in everyday activities at preschool – an exploratory study from Sweden

**DOI:** 10.3402/fnr.v60.32603

**Published:** 2016-10-06

**Authors:** Hanna Sepp, Karin Höijer

**Affiliations:** Food and Meal Science, School of Education and Environment, Kristianstad University, Kristianstad, Sweden

**Keywords:** preschool, teacher, meal education, food education, Sapere, cooking

## Abstract

**Background:**

There is a need for research both in relation to food education at preschools and in relation to how the individual teacher can handle and relate to the many different scientific facts and paradigms that are prevalent in relation to food, health, and a sustainable lifestyle.

**Objective:**

The aim of this study was to explore the experiences and meanings that preschool teachers associate with involving food as a tool for learning in planned educational activities.

**Design:**

An exploratory study was conducted in 14 preschools with 131 teachers. Twenty semi-structured individual or group interviews with 45 preschool staff were conducted, and 10 interviews were selected for analysis.

**Results:**

According to participants, both children and teachers developed a sensory language; children became more positive towards tasting and teachers discovered new possibilities for interdisciplinary work. However, the results also show that an allowing system, with both an interested and confident teacher who recognises the competent child and a supportive organisation, is needed in order to make food a meaningful tool for learning in preschool.

**Discussion:**

According to previous studies, food has the potential to play an important part in everyday activities at preschool, both in planned educational activities as well as at meal situations. Our results imply that a holistic understanding of food in preschool is required for long-term work with food as a natural part of the everyday activities.

**Conclusion:**

The results imply that it is fun and meaningful for both children and teachers, and quite possible, to work with food as a tool for learning in everyday activities at preschool. In order to include food as a way to work with the preschool curriculum for a sustainable lifestyle, an allowing system is needed.

According to the European Food Information Council (EUFIC), children's education concerning a healthy lifestyle is one of the most important keys to good health and to helping children reach their potential as the prevalence of health-related problems associated with food among children has increased during the last decades (1). Although food habits are not stable and do change during a person's lifetime, a base for healthy food habits can be established in early childhood (2). There are many preschool- and school-based interventions (aimed at decreasing obesity and increasing consumption of fruits and vegetables), systematically reviewed in several articles (3–10). Even though the studies have had different approaches, there is a clear common result; the effects of the interventions are limited. Common to the few cases where some change has actually been studied, is a collaboration of several different approaches, such as increased availability of fruits and vegetables, involvement of the home, as well as increased education on healthy food (3, 10, 11). This approach was, for example, employed in a comprehensive European study, the ToyBox study, which also pinpointed preschool teachers as key persons in obesity prevention work among children (12–14). In a recently published review by Mikkelsen et al. (8), 26 healthy-eating interventions in preschools were analysed. The review found that healthy-eating interventions in day-care facilities increased nutrition-related knowledge among the children as well as fruit and vegetable consumption. The conclusion was that preschools have the potential to influence children's food choice at an early age, which several other researchers also suggest (15–17) as well as the American Diet Association (18).

A great deal of learning about food and eating occurs early in life, especially regarding preferences for taste and attitudes towards food (19). As of 1998, Sweden has a specific curriculum for preschool with goals that specify the orientation of the work (20). The curriculum is comprehensive, and the expected developments are very detailed, but it does not specifically include food or meals, either from a health or a sustainable lifestyle perspective. In Sweden, most children aged 1–5 years are enrolled in preschool (21), where they have at least one meal per day and may consume as much as 70% of their daily energy and nutrient intake (22). The preschool and parents, thus, share the responsibility for children's food habits. Consequently, food, eating practices, and meals culture are experienced by young children both at home, in the private sphere, and at preschool, in the public sphere. Despite this, Swedish preschool teachers have very little theoretical and practical knowledge about preschool's role and responsibility for children's food habits (23).

Although, there are a lot of intervention studies which conclude that it is important to educate preschool children about food and health, little attention has been aimed at understanding preschools from the perspective of the teachers. There are several studies in the Nordic countries however, in which meals from a learning perspective are studied, for example in relation to gender (24), language skills (25–27), social interaction (28, 29), and care (30), but to our knowledge there is no research exploring conditions around food as part of planned educational activities in preschool either from a health perspective or from the perspective of teachers. This implies that there is a need for research both in relation to food education at preschools and in relation to how the individual teacher can handle and relate to the many different scientific facts and paradigms that are prevalent in relation to food, health, and a sustainable lifestyle. The aim of this study was to explore the experiences and meanings that preschool teachers associate with involving food as a tool for learning in planned educational activities.

## Methodology

The study design chosen to study teacher's experiences associated with involving food in planned educational activities was exploratory and had three stages. The first involved introducing participants to the study and to pedagogic methods. The second involved teachers working on their own by involving food in planned educational activities in their preschools during 1 year. The last involved data collection with qualitative interviews.

The first author was involved in a larger Leader project (31) called ‘The children's best table’ involving both preschools and schools with children aged 1–12. During fall 2012, county dietary managers and principals at these preschools were informed about the study of this article and invited to participate, and those who were interested enrolled. All teachers at these preschools were orally invited to participate in this study, and 125 decided to accept. In addition to these, one additional preschool was orally invited to participate and accepted, making the total number of participating preschools 14, with 131 teachers. All preschools were located in the south of Sweden.

In order to make it easier for the teachers to know what they can do with food, two books based on two specific pedagogic methods – Sensory education, the so-called Sapere (32), and Cook and learn step-by-step (33) – were introduced during the oral information. These were chosen based on availability and their self-instructive design. It was thus assumed that the participants did not need specific training to involve food in the planned educational activities. Sapere, which has proven to be a successful method (34–39), was developed for training and developing children's sensory abilities and language, where children are encouraged to experience food with all their senses and then put words to their experiences when they taste, smell, listen to, and touch different foods and meals. The step-by-step method was developed for baking and cooking with preschool children and means that children bake or cook something with the help of a sequence of cards with images and text (33, 40). What distinguishes the step-by-step method from other pedagogic image recipes is that only one portion is cooked; the children only make one scone or one smoothie. These methods gave the participants the opportunity to work with food as an educational instrument, with the help of concrete methods. Teachers at participating preschools worked with food in planned educational activities during 2013.

For this study, 20 semi-structured individual or group interviews with 45 preschool staff were conducted in 2014 during preschool hours, held in 10 different pre-schools, and all participants were women with a preschool-teaching degree. The interviews were conducted by the first author, who was trained in interview techniques and group interview methodology, as described by Patton (41). Interviews revolved around three themes about working with children in preschool: food in general at preschool, the curriculum in relation to food, and working with food in planned educational activities. Each teacher was interviewed on one occasion, either individually (for practical reasons) or in a group of 2–5 participants. The interviews lasted from 40 to 60 min and were video recorded.

The preliminary analysis began during the interview, and a pattern of teachers experiencing education with and about food as fun (possibilities) although hard to execute (barriers) emerged. Because saturation thus was experienced in the preliminary analysis of the interviews, a decision was made to transcribe verbatim one of two interviews per preschool, in total 10 group interviews with 23 teachers, using the qualitative software programme Transana, version 2.42. The transcripts were read through several times by both authors separately and sorted into codes using the analysis software Atlas.ti, version 7.5.

It is the experiences and the meanings that preschool teachers associate with involving food in their educational activities that are of interest in this article. Therefore, a social constructionist perspective was chosen because it provides an analytical framework through which it is possible to see how preschool teachers make sense of teaching about food. Wortham and Jackson (42) have described education as a ‘set of processes that occur in events and institutions that involve both informal socialization and formal learning’ (p. 107). This means that from a social constructionist perspective, education is a process in which various objects are (re)constructed, such as the role of the teacher. By adopting this theoretical perspective, it was possible to make visible how teachers make sense of teaching about food in relation to themselves, children, and preschool.

**Fig. 1:**
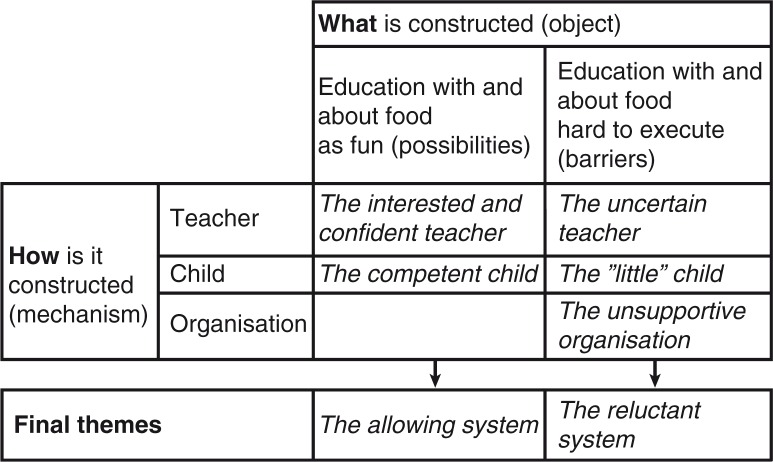
Matrix for social constructionist analysis of 10 interviews with 23 preschool teachers.

The codes were discussed and sorted into a matrix in which both the object that was being constructed (what) and the social relationship in which it was being constructed (how) were captured (42). According to Wortham and Jackson (42), an analysis presupposes, or assumes, that some things are solid. In the analysis of the preschool teacher interviews, it was apparent that the informants primarily made their experiences meaningful in relation to perceived barriers or possibilities. This meant that in the analysis, we assumed that the objects (barriers and possibilities) and mechanisms (teachers, children, and organisation) were stable aspects of the social world (see Fig. 1).

### Ethical considerations

The Ethical Regional Board at Lund University vetted the project protocol, and the requirements identified by the Swedish Research Council (2011) were followed. In addition to this, both selected pedagogical methods were based upon a foundational view that participation is voluntary and that all children have a right to their own taste and a right not to taste or participate.

## Results

Although the research questions for this study were focused on which experiences preschool teachers associate with involving food in *planned educational activities*, it soon became apparent that it was impossible for the preschool teachers to talk about food without bringing in the whole meal situation, that is, *all everyday activities* at preschool. Thus, the two main themes of the analysis, the allowing system and the reluctant system, should be understood in a broad perspective. In this section, we will explain these themes based upon the main categories.

### The allowing system

#### The interested and confident teacher

A majority of the participants did point out the possibilities and advantages of integrating food into educational activities as well as during meal times, mostly because it has been fun and because it has provided a new way of learning, even for the youngest. The methods have been experienced as inspiring, concrete, and easy to work with, and they have stimulated more cooking and baking with the children. The project made the teachers aware that they lack knowledge, but as they reflect on this they say that they've also learned a great deal. To use cooking as part of educational activities in preschool was new to most of them and has opened up new possibilities for interdisciplinary work.

Teacher: That you can use food for pedagogical purposes and have a lot of fun with different projects is to me a great benefit. And the joy of baking with children, well it doesn't have to be just baking, it can be something else that they appreciate, it's very, well to participate and help, they feel that they learn a lot. Many of them didn't know the measuring spoons at first, and there I've noticed a big change that they actually know now. They used to just say cup or something, and now it's ‘now we'll take this much’. They didn't use to know what it was or how much, and that it involves maths. For example you can tell them that this is maths – teaspoon, tablespoon and decilitre and things like that. We don't have to just do it with water as we used to; now we can do it with flour or sugar instead.

It appears as if the teachers hadn't considered preschool's potential to play an active role in working with food and good food habits before taking part in the project. However, it becomes apparent that preschool does have an important part to play in providing children with basic skills, and food habits are one part of this.

#### The competent child

The Sapere method's basic assumptions that all individuals have a right to their own taste and a right not to taste have been described as very positive starting points and have made the teachers reflect on their own part as role models and what rules they implement during regular meals. Many of the teachers also state that they learned a lot about their own taste and to put words to their experiences.

Another result is that the children are competent when it comes to food – more so than the teachers expected. The teachers have faith in the children's abilities but were still surprised at just how much the children were able to do themselves while they worked with the project.

Teacher: Just to have a recipe, that the child can interpret the recipe on its own is fantastic! They have learned that they can have a recipe to follow, that children can do things on their own. Because before we were the ones who decided that now we'll make buns and we need such and such. But here they can actually do it on their own and, as you say, the process changes completely.

Another effect of the project is that the children talk more about food. The Sapere method is described as a completely different way of working, giving the children the ability to put words to their experiences and develop a sensory language. Many teachers also mention the children's ability to say no and to justify why as a positive effect of the project. The children bring their new skills with them to the meal situations and are more positive towards tasting new things. Now the children consider what food tastes like and discuss with each other and have also become more conscious about and interested in food.

With the step-by-step method, the children have been given the opportunity to see and learn the whole process. The older children have helped the younger children, and they have all learned from each other. The children thought it was fun to cook and/or bake with the step-by-step recipes, and the teachers have perceived them as easy to read and follow. The results show that the teachers believe that food can be a means to gain understanding about other areas that can be hard to grasp, such as science.

### The reluctant system

#### The uncertain teacher

A majority of the participants did point out the possibilities and advantages of integrating food into educational activities; however, there were a few obstacles, for example, ignorance, either conscious or unconscious, and lack of interest that were constructed as absence of support. Conscious ignorance was, for example, when the teachers felt unsure about how they can work with food and children and experienced a lack of requisite skills and knowledge, which made them unable to turn a theory of methods into practice. Ignorance can also concern how the work should be organised or how many children you can work with in a group. Insecurity was also felt when they were unable to answer questions:

Teacher A: I had one [child] the other day who was thinking about ketchup and how ketchup was made or where the ketchup came from. We had ketchup that day and I read on the back and he [the child] noted that it was tomato and that they were crushed, and that he understood. So I read on the back what was in it and there was vinegar and some other things so you have to mix it. And then there was someone else who asked where the tomatoes came from and we agreed that it was from seeds. But before the first seed was found, where did they come from?

Teacher B: And then it became philosophy.

Teacher A: That's when I changed subject.

Unconscious ignorance was primarily connected to the Sapere method when they were to describe their experiences with the help of their senses; it was hard to verbalise these because they had never done so before. This lack of personal experience or practice led to a construction of the method as being slow and boring to work with.

Teacher: I think that adults find this hard too because when I have talked to other adults and explained what the children are saying, most of them think it is a bit funny. But it is actually quite hard to say if it is sweet or sour or bitter, we don't talk much about that. Many times you just say that something tastes good or not, not how. You don't describe taste very often, at least I don't.

A few informants expressed a lack of interest because they found it hard to see any benefits, stating that it needs enthusiasts with a significant interest to integrate food in the educational activities. None of the interviewed teachers mention that they have defined a purpose *before* the activities, and few reflect upon what the children have learned based on the goals of the curriculum; instead these were added in retrospect when compiling the documentation. The different parts of the curriculum were mentioned in general terms but also in the form of typical school subjects.

Teacher: Yes, there's a lot of maths in everything, it becomes integrated in all of it sort of. Chemistry and physics also become part of what you do.

The missing connection with the curriculum is especially apparent among the teachers who lack knowledge or interest concerning the integration of food into educational activities.

#### The ‘little’ child

The children's young age and limited experience were constructed as barriers by some informants. It is sometimes hard for them to remember, which makes it difficult to apply their knowledge to new situations. It can also be hard for the children to describe tastes and to have an individual taste preference; if one child says that a vegetable is bitter, other children tag along and say the same. The smallest children also generally lack language skills, while the older children lack the vocabulary to describe what they're experiencing in relation to food.

Teacher: We were tasting carrots. And it is hard for them, because they are not comfortable with seeing how it is; they are not comfortable with thinking about how they feel, with pausing to think about what they're doing. Because now we tasted the carrot when it was on a plate, when it was grated and when it was cut into smaller pieces, and they were given a carrot to nibble on, to see if they experienced any difference. It was new to them to approach it this way, to think and feel.

#### The unsupportive organisation

The most crucial barrier is the absence of a supporting organisation, both concerning pedagogical leadership and especially in the non-existing cooperation between the preschool units and the kitchens. Teaching staff and kitchen staff at all participating preschools had different managers, which means, for example, that regulations for purchases are unclear. The kitchens normally order their goods 2 weeks in advance, while the teachers make detailed plans of their activities weekly, making ordering ingredients via the kitchen difficult. Teachers also felt resistance from the kitchens to placing orders, because these purchases were charged to the kitchen's budget and not the unit's. In order to be able to work with the project, teachers at some preschools had to go shopping themselves or even bring the ingredients from their own homes.

Shortage of time was mentioned as an obstacle, both when working with documentation and preparations, and also that the children's young age called for extra time. The small number of staff was also mentioned in conjunction with the lack of time, which has meant that it is the teacher's own interest that decides whether or not they are going continue to work with food.

## Discussion

The results show that an allowing system, with both an interested and confident teacher who recognises the competent child and a supportive organisation, is needed in order to make food a meaningful tool for learning in preschool. Although there are several studies, in which meal situations from a learning perspective are studied (24–30), there is a lack of research exploring how food can be included as a key part in everyday activities at preschool in Sweden in a health and sustainable lifestyle perspective. Several studies (8, 15–17) and influential institutions as the EUFIC (1) and the American Diet Association (18) argues that children's education concerning a healthy lifestyle is an important key to good health. This study shows that food can be included as a key part in everyday activities at preschool, both in planned educational activities as well as at meal situations which means that both understanding and awareness of food in preschool is required.

Studies show that children dare to try more new foods and dishes when they have worked with sensory training (43, 44) and the Sapere method (34–38). This is in accordance with our results as well as a recently published dissertation from the Netherlands (39), where both teachers and children (4–12 years) experienced the laboratory learning with sensory exercises as very positive. The study revealed that children's knowledge about healthy eating habits increased with the help of sensory training – especially in combination with other methods of cooking and field trips. However, our result shows that there is a need for an allowing system in order to make children's education concerning food habits and a sustainable lifestyle feasible and meaningful on a long-term basis. For example, teachers experienced the lack of a supportive organisation, both concerning pedagogical leadership and in cooperation with the kitchen staff. From this, it can be concluded that a supportive manager with clear leadership and a learning organisation is necessary to efficiently plan activities, and our results are in line with Altin et al. (45) who have studied and evaluated the criteria surrounding the implementation process of health promotion preschool environment in a Swedish municipality.

The curriculum does not specifically include food or meals as a tool for learning, either from a health or a sustainable lifestyle perspective. Research implies that even though the Swedish preschool has had a specific curriculum (20) for almost 20 years there is still a need to transform the curriculum into an active document (46, 47). This became apparent in this study when teachers planned and carried out activities involving food without using the curriculum as a supportive framework but rather as a ‘hindsight document’. The teachers in this study reported that they usually have confidence in the children's ability, which is in line with the curriculum as well as Swedish research (e.g. 47). Despite this, the teachers were challenged by the step-by-step method which assumes that children can manage all the steps on their own. Why is that so? Cooking almost always involves an expectation of an edible result, which could create uncertainty in the teacher relating to the children's performance, and it can be assumed that this uncertainty primarily has to do with the teacher's own lack of didactic knowledge and food-related skills.

Educational theory assumes that children learn from and with each other, which no teacher in this study seems to have considered in relation to food before, but which became obvious during the project. This result surprised us. Is it because food and meals are an everyday activity, a routine situation, where teachers haven't really thought about food in relation to their profession? This implies a need for a holistic approach to understand and work with food in preschool.

### Methodological considerations

A total of 20 interviews were conducted, either individually or in groups of 2–5 participants. Patton (41) states that group interviews generate high-quality and data-intense material, which also was observed in this study, as the individual interviews were less rich. Thus, to maintain rigor in the material, only group interviews were selected for the final analysis.

There is a shortage of educational material related to food in preschool, and therefore, Cook and learn step-by-step was chosen (33). The Sapere method (32) was chosen because it has proven to be a successful method (34–38). Because the books are self-explanatory, we assumed that there was no need for training in methods. Judging by the result, we can retrospectively see that we should have educated the teachers about the methods so they would feel secure. Moreover, even the kitchen staff should have been included, even though the study focused on the planned educational activities, since it became apparent that it was impossible for the preschool teachers to talk about food without bringing in the whole meal situation, which involves the kitchen staff very much.

For this study, we chose a social constructionist perspective for analysis of the material because the intention was to gain insight into the experiences of preschool teachers with a focus on food as part of their everyday activities, rather than on processes of learning and teaching or which pedagogic perspectives different preschools work with. Therefore, we believe that a social constructionist perspective was an adequate approach to process the results.

### Implications for research and practice

To include food as an important part in everyday activities at preschool, a holistic understanding of food in preschool is required for long-term work. The lack of experienced individual and/or structural support makes it hard to integrate food as a tool for leaning as a natural part of everyday activities. There is no potential at all to work with food if the system is reluctant. However, if either individual or structural support is lacking, it may still be possible to work with food during a limited time, as was the case with the reported project. To enable long-term work, with food as a natural part of the everyday activities and with food as a way to work with the preschool curriculum for a sustainable lifestyle, an allowing system is needed. The preschool teachers need knowledge, skills, and tools, as well as the support of managers concerning both organisation and the allocation of resources. Otherwise, the success of implementing food as a tool for learning relies on enthusiasts who consider it important that food should be integrated as a natural part of preschool.
